# Relationship between Age and the Ability to Break Scored Tablets

**DOI:** 10.3389/fphar.2016.00222

**Published:** 2016-07-26

**Authors:** Kim Notenboom, Herman Vromans, Maarten Schipper, Hubert G. M. Leufkens, Marcel L. Bouvy

**Affiliations:** ^1^Department of Public Health Effects, National Institute for Public Health and the EnvironmentBilthoven, Netherlands; ^2^CBG, Medicines Evaluation BoardUtrecht, Netherlands; ^3^Department of Pharmaceutical Sciences, Faculty of Science, Utrecht UniversityUtrecht, Netherlands; ^4^Department of Clinical and Compounding Pharmacy, University Medical Centre UtrechtUtrecht, Netherlands; ^5^Department of Statistics, Computer Science and Modelling, National Institute for Public Health and the EnvironmentBilthoven, Netherlands; ^6^SIR Institute for Pharmacy Practice and PolicyLeiden, Netherlands

**Keywords:** tablet breaking, score line, age, older adults, comparative study

## Abstract

**Background:** Practical problems with the use of medicines, such as difficulties with breaking tablets, are an often overlooked cause for non-adherence. Tablets frequently break in uneven parts and loss of product can occur due to crumbling and powdering. Health characteristics, such as the presence of peripheral neuropathy, decreased grip strength and manual dexterity, can affect a patient's ability to break tablets. As these impairments are associated with aging and age-related diseases, such as Parkinson's disease and arthritis, difficulties with breaking tablets could be more prevalent among older adults. The objective of this study was to investigate the relationship between age and the ability to break scored tablets.

**Methods:** A comparative study design was chosen. Thirty-six older adults and 36 young adults were systematically observed with breaking scored tablets. Twelve different tablets were included. All participants were asked to break each tablet by three techniques: in between the fingers with the use of nails, in between the fingers without the use of nails and pushing the tablet downward with one finger on a solid surface. It was established whether a tablet was broken or not, and if broken, whether the tablet was broken accurately or not.

**Results:** The older adults experienced more difficulties to break tablets compared to the young adults. On average, the older persons broke 38.1% of the tablets, of which 71.0% was broken accurately. The young adults broke 78.2% of the tablets, of which 77.4% was broken accurately. Further analysis by mixed effects logistic regression revealed that age was associated with the ability to break tablets, but not with the accuracy of breaking.

**Conclusions:** Breaking scored tablets by hand is less successful in an elderly population compared to a group of young adults. Health care providers should be aware that tablet breaking is not appropriate for all patients and for all drugs. In case tablet breaking is unavoidable, a patient's ability to break tablets should be assessed by health care providers and instructions on the appropriate method of breaking should be provided.

## Introduction

Pharmaceutical care becomes more complicated with advanced age because the characteristics and health problems of older adults people are different and often more complex than those of young adults. Current incentives to optimize pharmacotherapy in the geriatric population include reducing inappropriate prescribing and improving medication adherence (Hill-Taylor et al., 2013; Fastbom and Johnell, 2015; Ni Chroinin et al., 2015; Scott et al., 2015; Projovic et al., 2016). Practical problems that hinder older patients to use their medicines correctly, such as difficulties opening packaging, swallowing medicines or breaking tablets, are an often overlooked cause for non-adherence. However, these problems can lead to incorrect use of medicines with clinically relevant consequences (Notenboom et al., 2014).

Several studies have shown that patients experience breaking of scored tablets a difficult or painful task (Rodenhuis et al., 2003; Denneboom et al., 2005; Quinzler et al., 2007; Notenboom et al., 2014). Tablets frequently break in uneven parts and loss of product can occur due to crumbling and powdering, which impedes the accuracy of dosing (Spang, 1982; Gupta and Gupta, 1988; McDevitt et al., 1998; Wilson et al., 2001; Verrue et al., 2011; Helmy, 2015). At the same time, tablet breaking is common practice, with an estimated frequency in primary care at 24–31% (Rodenhuis et al., 2004; Quinzler et al., 2006). Characteristics of a tablet, such as size, shape, hardness, and one- or two-sided presence of the score line, have an impact on how easy a tablet can be broken (Spang, 1982; van Santen et al., 2002; van der Steen et al., 2010). Furthermore, the method of breaking can affect the ease and accuracy of breaking (Wilson et al., 2001; Van Vooren et al., 2002). Health characteristics, such as the presence of peripheral neuropathy, decreased grip strength and manual dexterity, or vision problems can influence a patient's ability to break tablets. As these impairments are associated with aging and age-related diseases, such as Parkinson's disease and arthritis, difficulties with breaking tablets could be more prevalent among older adults compared to young adults. Concurrently, elderly people are more often in need of scored tablets, as they often require a lower dose strength compared to young adults. These lower strengths are not always available (Rodenhuis et al., 2004).

Little is known about the ability of older adults patients to break scored tablets manually. Findings are contradictory, and previous studies evaluated only one or two tablets, allowed the use of splitters or did not address breaking methodology at all (McDevitt et al., 1998; Pautas et al., 2011). Therefore, the objectives of this study were to investigate the relationship between age and the ability to break a large sample of scored tablets by three manual techniques for breaking tablets.

## Materials and methods

### Study design

A comparative study design was chosen. Thirty-six older adults and 36 young adults were systematically observed with breaking 12 different, scored tablets, each tablet by three common techniques for breaking tablets by hand.

### Participants

The older people were recruited in five residential homes for elderly in the area of Utrecht, the Netherlands. People were eligible if they were aged 65 and older and managed their own medication. Exclusion criteria were dementia, blindness and impaired use of hands and/or fingers. These criteria derive from a test procedure to assess the ability of older people to break scored tablets, which was developed in a previous study (Barends et al., 2005). Employees of the residential homes approached eligible people and explained the purpose of the study. When interested, they were given an information letter that included more detailed information about the study. After a week, approached people were asked whether they wanted to participate in the study. One person was excluded by the researchers at the start of the study because of temporarily impaired use of hands. Four individuals dropped out during the first day of the study: two due to loss of interest, one because of too much pain in the shoulder during the breaking of the tablets, and the fourth person found the study too intensive. Excluded and dropped out individuals were replaced.

The young adults were recruited among Master students from the School of Pharmacy at Utrecht University, the Netherlands. Participation of the young adults was on voluntary basis as part of a study course. All 36 approached young adults agreed to participate and finalized the study.

The study was not subject to The Medical Research Involving Human Subjects Act (WMO). The study was conducted in compliance with the requirements of the UPPER institutional review board (http://www.uu.nl/vkc/upper). For this type of study, informed consent is not required in the Netherlands.

### Tablets

Twelve commercially available scored tablets were selected for this study: four different active pharmaceutical ingredients, and three different brands of each: bisoprolol 5, citalopram 20, enalapril 5, and paroxetine 20 mg (coded A1-3, B1-3, C1-3, and D1-3, respectively). The criteria for selection of the tablets were presence of a score line intended for subdivision into equal doses and common use in the geriatric population. At least one of the brands of each active pharmaceutical ingredient had a pressure sensitive score line. The tablets differed in size, shape, and score-line characteristics (**Figure 3**).

### Sample size

Sample size was calculated based upon data generated by a previous study on the ability of older adults to break tablets. It was found that older people were able to break 74% of the tablets, taking the multiple measurements within each participant into account (Barends et al., 2005; van der Steen et al., 2010). With a type one error (α) for a one-sided test of 0.05 and a power of 80%, it was found that 10 participants were needed in each of the two age groups to demonstrate a difference of 15% in the ability to break tablets, again taking the multiple measurements within each participant into account. As we aimed to investigate several potential determinants, we decided to include a convenience sample of 36 participants per age group.

### Data collection and measures

**Figure 1:**
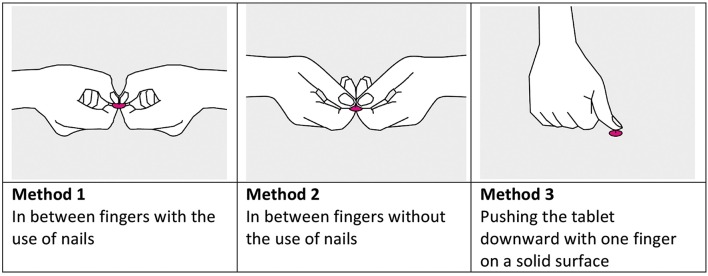
**The three methods used in the study for breaking scored tablets by hand**.

The 12 tablets were broken by each participant, each tablet by three common techniques for breaking tablets by hand: breaking in between the fingers with the use of nails, breaking in between the fingers without the use of nails and pushing the tablet downward with one finger on a solid surface (Figure 1). The participants received a written and a verbal explanation of these three techniques. Tablet breaking was spread over 2 days for each participant and a 3-h break was implemented halfway on each day. The tablets were presented to the participants in a random order to minimize the possible effects of “training in breaking” and “getting tired after some acts of breaking.” Participants' age and sex, and experience with tablet breaking were collected.

The primary outcome measures were the ability of the participants to break the tablets and the ability of the participants to break the tablets in equal halves, i.e., the accuracy of breaking. To determine the ability of breaking, it was established whether a tablet was broken or not. Tablets were scored as “broken” regardless of the outcome of breaking, e.g., broken in two halves, in three of more fractions, crumbled or powdered upon breaking. The accuracy of breaking was determined for each broken tablet based upon the mass deviation of the obtained tablet parts from the theoretical halved weight of the parent tablet. A deviation of not more than 15.0% from the theoretical halved weight of the parent tablet was allowed. This criterion was derived from the European Pharmacopeia (European Pharmacopoeia, 2013). Only tablets for which both halves complied with the criterion were scored as “accurately broken.” When tablets broke into quarters, two quarters were combined and treated as halves. In case the quarters remained attached by the coating layer, the attached parts were considered as halves. In all other situations, e.g., when tablets were broken into three, five or more parts or completely crumbled upon breaking, the tablet was considered as exceeding the permitted deviation and scored as “not accurately broken” (Barends et al., 2006). Tablets were weighed individually and placed in a separate numbered and coded bag prior to breaking. After breaking, the resultant portions were returned to the same bag. The tablets and obtained tablet parts were weighed to the nearest 0.0001 g (Mettler Toledo AT201 analytical balance).

### Data analysis

Participants' characteristics are shown as mean and % (n). The aggregated results for the ability and accuracy of breaking for each age group are reported as relative frequencies. The proportions of broken tablets and accurately broken tablets were compared between groups by independent *t*-tests.

The relationship between age and the ability to break tablets, and the relationship between age and the ability to break tablets accurately was further evaluated by mixed-effects logistic regression modeling. Besides age, the fixed variables of interest were gender, method of breaking and tablet. Gender was included because the stronger grip strength of men can potentially influence their ability and accuracy of tablet breaking. Method of breaking and tablet characteristics are known to influence the ease and accuracy of tablet breaking. Because the data visualization revealed a relation between type of score line and method of breaking, the interaction between tablet and method of breaking was added. Each model included a random intercept for the participants to account for within-participant correlation. Six models were fit to the data. Model 1 examined the relation between the ability or accuracy of breaking and age. Next, the explanatory variables were added to the first model. Model 2 included age and gender, Model 3 included age, gender and method of breaking, Model 4 included age, gender and tablet, Model 5 included age, gender, tablet and method of breaking, and Model 6 included age, gender, tablet, method of breaking and the interaction between tablet and method of breaking. The preferred model was selected using the Akaike information criterion (AIC). Effect estimates were reported as odds ratios (ORs), along with 95% confidence intervals (CIs). The discriminative ability of the model was assessed with the *c*-index (i.e., the area under the ROC curve). Statistical tests were two-sided, and significance was set at *P* < 0.05. The *t*-tests were conducted using SPSS Statistics, version 22 (IBM SPSS), and R programming language version 3.2.2 was used for modeling (http://www.R-project.org/).

## Results

The mean age of the 36 older participants was 84.2 years, and 69.4% were women. The mean age of the 36 young participants was 24.8 years, and 80.6% were women. Among the older participants, 22.2% was experienced with breaking tablets. None of the young participants was experienced with tablet breaking. The participants' characteristics are presented in Table 1.

**Table 1:** **Characteristics of the participants**.

**Characteristics^a^**	**Elderly people (*n* = 36)**	**Young adults (*n* = 36)**
Mean [SD] age, years	84.2 [6.8]	24.8 [1.8]
Women	69.4% (25)	80.1% (29)
Experienced with breaking tablets	22.2% (8)	0% (0)

a The information in this table is presented as % (n) unless otherwise indicated.

Each participant attempted to break a total of 36 tablets; 12 tablets by three different methods of breaking. Compared to the young participants, the ability of the older participants to break the tablets was significantly lower. On average, the older adults broke 38.1% of the 36 tablets and the young adults broke 78.2% of the 36 tablets (*P* < 0.001). There was no statistically significant difference in the mean proportion of accurately broken tablets between the two age groups; the older adults broke on average 71.0% of the broken tablets accurately, whereas the young participants broke 77.4% of the broken tablets accurately (*P* = 0.116). Although, not our primary objective, we also compared the outcomes between the genders. On average, the proportion of tablets broken by male participants was significantly higher compared to the proportion broken by women (67.0 and 55.2%, respectively, *P* = 0.035). This trend was observed in both age groups, although the difference was not significant among the young adults. Contrarily, the mean proportion of accurately broken tablets was lower for male participants compared to female participants (68.5 vs. 76.1%; *P* = 0.109). This difference was not significant.

There were no statistically significant differences in the proportion of tablets broken and the proportion of tablets broken accurately between older participants with and without experience in breaking tablets (38.5 vs. 38.0%; *P* = 0.951, and 69.3 vs. 71.4%; *P* = 0.813, respectively).

**Figure 2:**
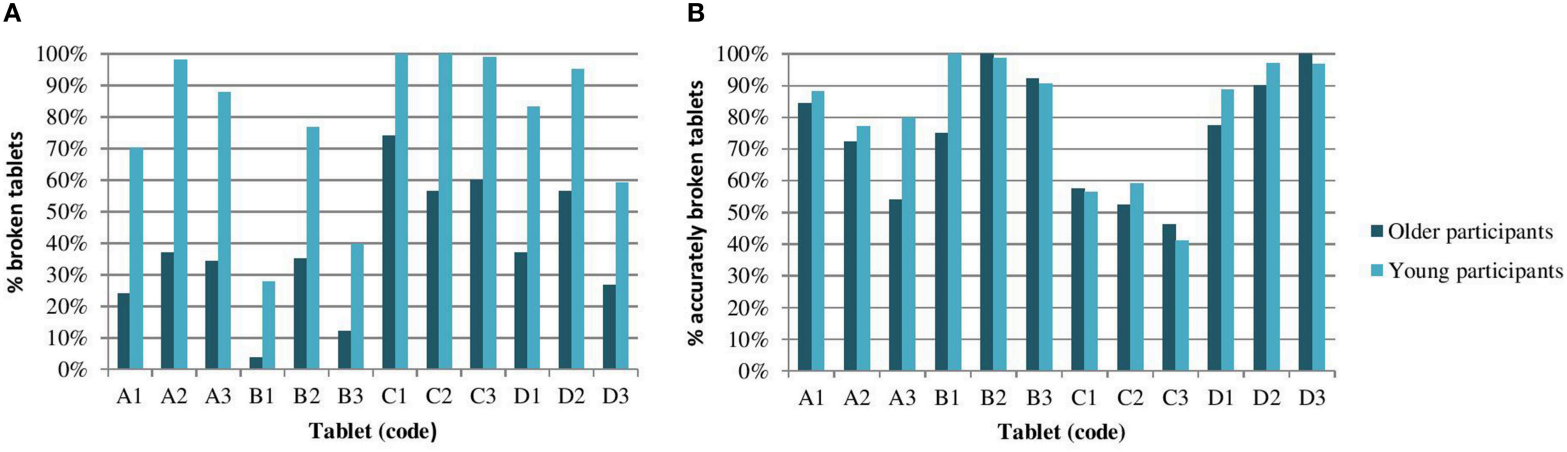
**The results for ability and accuracy of breaking, for tablets A1-D3 individually**. The percentage of tablets broken by the older and young participants **(A)**, and the percentage of accurately broken tablets, of those that were broken **(B)**.

The results for the individual tablets, as visualized in Figure 2, showed that for each of the 12 tablets the proportion of tablets broken by the older participants was considerably lower compared to the proportion broken by the young participants. For each individual tablet, no clear difference between the age groups was observed for the proportion of accurately broken tablets. Both the ability of breaking and the accuracy of breaking showed a high inter-tablet variability, which appeared similar between the two age groups. The proportion of tablets broken by the older participants ranged between 3.7% (tablet B1), and 74.1% (tablet C1), whereas the proportion of tablets broken by the young participants ranged between 27.8% (tablet B1), and 100% (tablet C1, C2).

**Figure 3:**
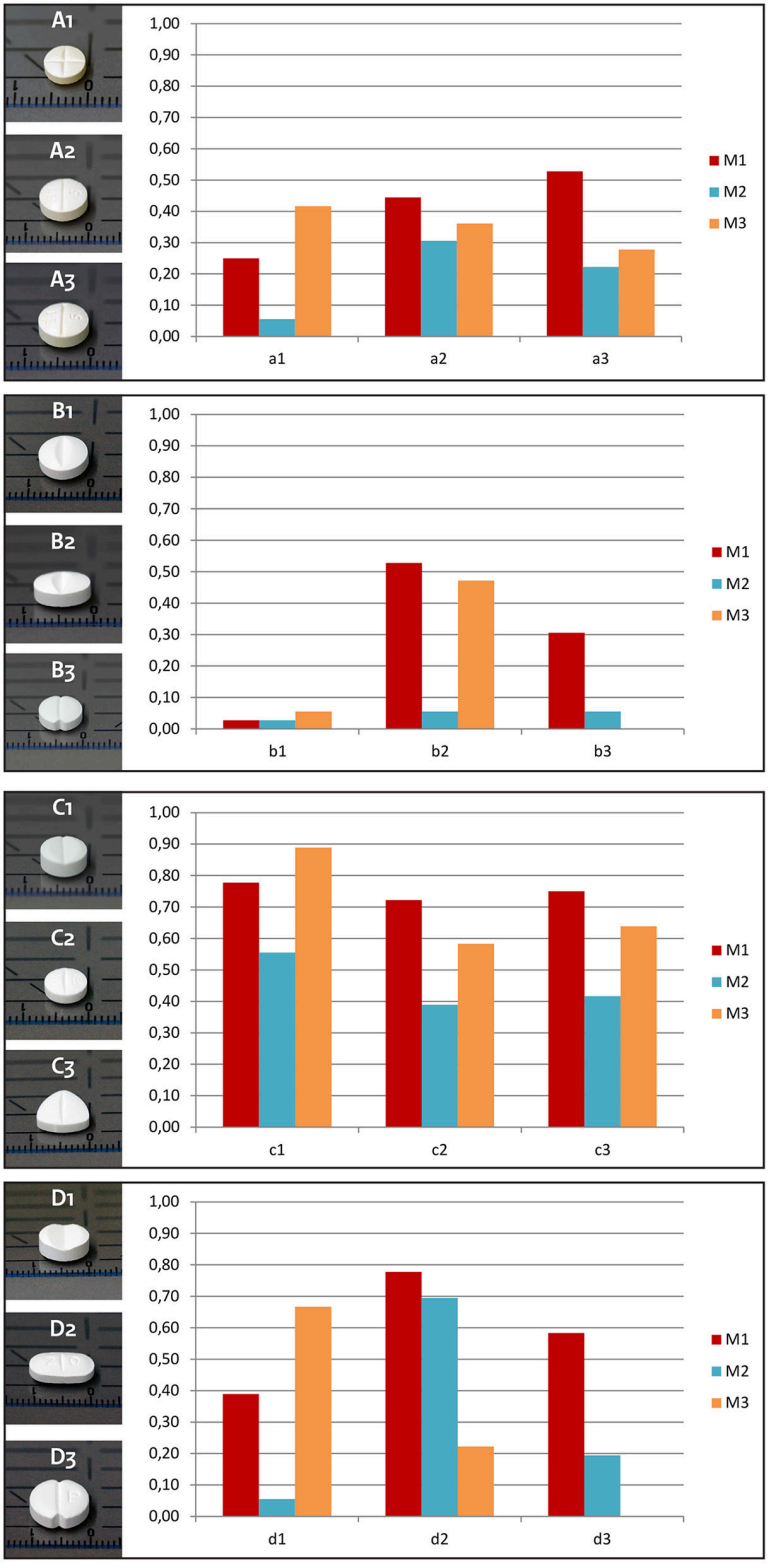
**Proportion of tablets that is broken by the older participants by the three methods of breaking**. M1 = Breaking in between the fingers, with the use of nails. M2 = Breaking in between the fingers, without the use of nails. M3 = Breaking by pushing the tablet downward with one finger one a solid surface.

Figure 3 shows the ability of the older participants to break each of the 12 tablets by the three breaking techniques. The tablets with a pressure sensitive score line, i.e., tablets A1, B1, C1, and D1, were easier to break by pushing them downward on a hard flat surface. All other tablets were easier to break between the fingers, with the use of nails.

The relationship between age and the ability and accuracy of tablet breaking was further analyzed by mixed-effects logistic regression modeling. According to the AIC, the most complex model (Model 6) best explained the ability of breaking between participants (Table 2; *OR* = 50.56, 95% *CI* = 25.02–108.03, *P* < 0.001). Model 6 also best explained the accuracy of breaking. However, age was not significantly related to the accuracy of breaking (Table 3: *OR* = 1.19, 95% *CI* = 0.81–1.75, *P* = 0.364). The other determinants gender, tablet, and method of breaking were significant for both the ability and accuracy of breaking scored tablets. The *c*-indexes of these models were 0.945 and 0.851, respectively, meaning that the models' ability to discriminate between tablets that break or do not break (accurately) is very good.

**Table 2:** **Odds ratios (ORs) and model characteristics for the ability of breaking**.

**Models**	**Age Reference: Older participants**		**Gender Reference: Female participants**		**AIC**
	**OR (95% CI)**	***P*-Value**	**OR (95% CI)**	***P*-Value**	
Model 1: Random effect for participant; fixed effect for age	7.23 (4.76;11.17)	<0.001	–	–	2920.5
Model 2: Model 1 + fixed effect for gender	7.93 (5.44;11.78)	<0.001	2.5 (1.62;3.85)	<0.001	2907.01
Model 3: Model 1 + fixed effects for gender and method of breaking	8.64 (5.83;13.05)	<0.001	2.59 (1.65;4.07)	<0.001	2823.41
Model 4: Model 1 + fixed effects for gender and tablet	19.38 (11.31;34.12)	<0.001	3.59 (1.96;6.57)	<0.001	2213.05
Model 5: Model 1 + fixed effects for gender, method of breaking and tablet	24.22 (13.56;44.76)	<0.001	3.95 (2.06;7.57)	<0.001	2090.54
Model 6: Model 5 + interaction between method of breaking and tablet	50.56 (25.02;108.03)	<0.001	4.99 (2.28;10.9)	<0.001	1809.76

CI, confidence interval; OR, odds ratio; AIC, aikake information criterion.

**Table 3:** **Odds ratios (ORs) and model characteristics for the accuracy of breaking**.

**Models**	**Age Reference: Older participants**		**Gender Reference: Female participants**		**AIC**
	**OR (95% CI)**	***P*-Value**	**OR (95% CI)**	***P*-Value**	
Model 1: Random effect for participant; fixed effect for age	1.45 (1.06;1.97)	0.015	–	–	1676.77
Model 2: Model 1 + fixed effect for gender	1.31 (0.97;1.76)	0.074	0.65 (0.48;0.87)	0.005	1671.54
Model 3: Model 1 + fixed effects for gender and method of breaking	1.35 (1;1.82)	0.044	0.65 (0.48;0.88)	0.005	1668.2
Model 4: Model 1 + fixed effects for gender and tablet	1.1 (0.76;1.57)	0.605	0.52 (0.35;0.75)	0.001	1368.25
Model 5: Model 1 + fixed effects for gender, method of breaking and tablet	1.13 (0.79;1.61)	0.515	0.52 (0.35;0.75)	0.001	1368.99
Model 6: Model 5 + interaction between method of breaking and tablet	1.19 (0.81;1.75)	0.364	0.51 (0.34;0.77)	0.001	1315.94

CI, confidence interval; OR, odds ratio; AIC, aikake information criterion.

## Discussion

The present study investigated the relationship between age and the ability to break scored tablets by hand, as well as between age and the accuracy of the same. Our findings demonstrate that older adults more frequently experience difficulties breaking scored tablets than young adults. Moreover, older adults were considerably less able to break tablets compared to young adults (OR = 50.56, *P* < 0.001). Contrary to the ability of breaking, it was found that age was not related to the accuracy of breaking (OR = 1.19, *P* = 0.364).

The findings of this study further show that a persons' ability to break a tablet is not only attributable to advanced age. Gender, the tablet itself and the method of breaking also contribute to an individuals' ability to break a tablet. To our knowledge, the effect of gender on the ability of breaking by hand was not identified before. In contrary, studies that allowed breaking by using a knife showed that gender was not predictive for the accuracy of breaking (McDevitt et al., 1998; Zaid and Ghosh, 2011). Our finding that the ability and accuracy of breaking are influenced by the type of tablet, i.e., the physical characteristics of the tablet, confirms the findings of several other studies. The older participants most easily broke tablets C1–3 and D2. Tablets C2, C3 are the thinnest tablets among our sample, with the exception of tablet A1. Previous studies showed that that thinner tablets are easier broken than thicker ones (Spang, 1982; van Santen et al., 2002). Although, tablet A1 was the thinnest tablet, it was also the tablet with the smallest diameter (5.7 mm) and therefore more difficult to handle, especially for the older participants. Tablet D2 is oblong shaped and has the largest diameter (11.6 mm) of our sample. Previous studies showed that oblong tablets are more easily broken than round ones, and that oblong tablets should have a diameter not smaller than 10 mm to be sufficiently breakable (van Santen et al., 2002; van der Steen et al., 2010). A few studies showed the impact of the manual technique of breaking, although for only one or two tablets (Wilson et al., 2001; Van Vooren et al., 2002). The relation between the characteristics of a tablet and the method of breaking was however not addressed before.

The decreased ability of older adults to break tablets could be explained by a reduction in handgrip strength with advanced aging. This is supported by the finding that male participants broke more tablets compared to women, as men are known to have stronger grip strength than women (Budziareck et al., 2008; Incel et al., 2009). The absence of a relationship between age and the accuracy of breaking suggests that the accuracy of breaking is less affected by grip strength. Moreover, McDevitt et al. found that grip strength of men was inversely associated with the accuracy of tablet breaking (McDevitt et al., 1998).

### Implications for clinical practice

Problems with tablet breaking are not just a convenience issue. The occurrence of these problems will add to the regimen complexity, increasing the risk for non-adherence, medication errors and adverse drug reactions. The high prevalence of difficulties with breaking scored tablets observed in this study, stresses the need to diminish the occurrence of this problem. Manufacturers should avoid the use of score lines that are intended for dose adjustment, e.g., by producing tablets with dose strengths that correspond to the lower geriatric dose recommendations. In those situations where the presence of a score line is justified, manufacturers should validate the claimed functionality of the score line by breakability testing conducted in a population representative for the people that will break the tablet in daily practice. To date, the pharmacopoeial standards for the assessment of the performance of score lines do not define characteristics of the person performing the test (European Pharmacopoeia, 2013; The International Pharmacopoeia, 2015). Furthermore, tablets may also contain a score line to facilitate swallowing instead of breaking in equal halves for dosing purposes. There are no regulatory requirements for these score-lines. The observed decreased ability of the older adults to break tablets that are scored for dosing purposes raises also a concern about the functionality of score lines intended to facilitate swallowing. It should be considered to assess the functionality of these score lines too.

From a patient perspective, health care providers could take an active role in improving therapeutic outcomes and reducing adverse consequences due to inaccurate dosing by addressing potential difficulties with breaking. Pharmacists should evaluate a person's ability to break a tablet accurately and determine the most suitable method of breaking. This should be done for each drug and each patient. In situations where a patient is not able to break the prescribed tablet, other solutions should be looked for. A different brand of the same product or a different dosage form could be more appropriate. When no alternatives are available, therapeutic substitution with an alternative that is available in an appropriate strength may sometimes be an option. Also, the tablets could be dispensed in equal halves by the pharmacy or another dosage form such as capsules could be compounded. Stability of the broken tablets should than however be guaranteed. This point could be addressed by drug product manufacturers.

As the ability of breaking is influenced by tablet characteristics, it is relevant that a persons' ability to break the prescribed tablet is re-evaluated when generic substitution or other brand dosage changes take place. Attention should be paid on any change in score line type, and therewith on instructions on the appropriate method of tablet breaking. Currently, information on the score line type, i.e., pressure sensitive or not, and instructions on how to break the tablet are not always present in the product information. For the 12 tablets investigated during this study, the patient information leaflet of only one tablet (A1) included an instruction on how to break the tablet. It is recommended that the instructions on the appropriate method of tablet breaking become a mandatory part of the patient information leaflet for tablets with a score line.

### Limitations

Our study has some limitations. It could be argued that the selection of participants from homes for elderly is not representative of community dwelling older adults. However, the people selected were living in either sheltered or so-called service accommodation, and were not eligible for help with the use of their medication. They all managed their own medication. The inclusion and exclusion criteria for the older adults were chosen to compile a “worst case” group of people that is required to break tablets for dosing purposes in daily practice. Likewise, the young adults represent a “best case” group. These two age groups represent both ends of the Gaussian distribution. Even among the young adults the results of breaking were not fully satisfactory.

The participants did not have to subdivide the tablets included in our study on a daily basis. Patients might overcome their difficulties when they get more familiar and experienced with the breaking of a certain tablet. On the other hand, in many countries generic substitution may take place more than once during a year, reducing the effect of training by breaking.

We might have unobserved confounding. The two groups of volunteers might differ not only with respect to the observed characteristics like age and sex, but also with respect to unobserved characteristics like frailty, finger size, grip strength etc., that might influence the outcome.

We did not investigate the breaking of unscored tablets and neither did we include the use of aids, such as kitchen knives or tablet splitters, although both are used in practice. Breaking unscored tablets is considered unlicensed use, and the result is expected to be worse compared to breaking of scored tablets. Additionally, the basic principle should be that patients do not need aids, such as splitting devices or knives to obtain the prescribed dose from scored tablets. Several studies suggest that these aids do not accurately halve tablets (Peek et al., 2002; Polli et al., 2003; Hill et al., 2009; Shah et al., 2010; Verrue et al., 2011; van Riet-Nales et al., 2014). In addition, patients may harm themselves using knives. The risk on harm may even be increased in patients who have impaired manual function, which is often the reason why they are not able to break tablets manually.

To our best knowledge, this is the first study to demonstrate the relationship between age and the ability to break scored tablets by hand. Furthermore, we included three manual techniques of breaking and a relatively high number of tablets with different characteristics compared to many other studies.

## Conclusions

This study demonstrates that the breaking of scored tablets by hand was less successful in a population of older adults compared to a group with young adults. Health care providers should be aware that tablet breaking is not appropriate for all patients and for all drugs. To ensure safe self-management of medicines, breaking tablets should be avoided in older patients and the use of alternatives should be considered. In case tablet breaking is unavoidable, health care providers should asses a patient's ability to break tablets and provide instructions on the appropriate method of breaking.

## Author contributions

KN was involved in study design, data collection, data analyses, interpretation of the results, and manuscript writing. MS was involved in data analyses and manuscript writing. HV, MB, and HL were involved in study design, interpretation of the results and manuscript writing. All authors and the RIVM review board approved the final manuscript.

### Conflict of interest statement

KN, HV, MS, and MB declare no conflicts of interest. HL declares that no direct funding or donations from private parties, including the pharmaceutical industry, have been received. Research funding from public-private partnerships, i.e., IMI and TI Pharma has been accepted under the condition that no company-specific product or company related study is conducted. Unrestricted research funding from public sources, i.e., The Netherlands Organization for Health Research and Development (ZonMW), the EU 7th Framework program (FP7), the Dutch Medicines Evaluation Board (MEB), the National Health Care Institute (ZIN), and the Dutch Ministry of Health, Welfare and Sport.
